# Clinical outcomes and in vivo wavefront analysis of a toric transcleral plug-fixated intraocular lens using a pyramidal sensor aberrometer

**DOI:** 10.1097/j.jcrs.0000000000001930

**Published:** 2026-03-03

**Authors:** Lorenzo de Angelis, Vito Romano, Fabrizio Giansanti, Gianni Virgili, Marianna Della Porta, Stanislao Rizzo, Francesco Barca

**Affiliations:** From the University of Florence, Florence, Italy (de Angelis); Department of Ophthalmology, Palagi Hospital, Azienda USL Toscana Centro, Florence, Italy (de Angelis, Barca); Department of Medical and Surgical Specialties, Radiological Sciences, and Public Health, University of Brescia, Brescia, Italy (Romano); Department of Neurosciences, Psychology, Drug Research, and Child Health, Eye Clinic, University of Florence, AOU Careggi, Florence, Italy (Giansanti, Virgili, Della Porta); Ophthalmology Unit, Fondazione Policlinico Universitario A. Gemelli IRCCS, Rome, Italy; Catholic University “Sacro Cuore,” Rome, Italy; Neuroscience Institute, Italian National Research Council (CNR), Pisa, Italy (Rizzo).

## Abstract

**Purpose:**

To evaluate the visual, refractive, and aberrometric outcomes of a toric (T) FIL–SSF intraocular lens (IOL) implanted in aphakic eyes lacking capsular support.

**Setting:**

Ospedale Piero Palagi and Azienda Ospedaliera Universitaria Careggi, Florence, Italy.

**Design:**

Multicenter prospective observational case series.

**Methods:**

Aphakic eyes with regular corneal astigmatism >1 diopter (D) underwent sutureless transscleral fixation of a T FIL–SSF IOL. Preoperative assessment included uncorrected distance visual acuity (UDVA), corrected distance visual acuity (CDVA), manifest refraction, corneal tomography, and biometry. Postoperative visits occurred at 1 day; 7 days; and 1 month, 3 months, and 6 months. At 6 months, vector astigmatism analysis, wavefront aberrometry, and IOL alignment were performed.

**Results:**

15 eyes from 12 patients completed the 6-month follow-up. Mean UDVA improved from 0.96 ± 0.25 to 0.19 ± 0.10 logMAR and CDVA from 0.41 ± 0.32 to 0.04 ± 0.05 logMAR (both *P* < .01). Mean postoperative spherical equivalent prediction error was −0.12 ± 0.54 D. Mean absolute corneal astigmatism decreased from 2.15 ± 0.54 D to a mean refractive astigmatism of 0.73 ± 0.48 D. Wavefront aberrometry showed total astigmatism of 0.36 ± 0.28 µm, with mean total higher-order aberrations of 0.24 ± 0.08 µm. Mean IOL misalignment was 3.33 ± 3.42 degrees, significantly correlated with residual refractive astigmatism (ρ = 0.71 *P* < .01).

**Conclusions:**

T FIL–SSF IOL implantation provided substantial improvements in visual acuity, predictable refractive outcomes, and significant cylinder reduction in aphakic eyes with corneal astigmatism lacking capsular support.

## Abstract 2

Implantation of a toric FIL–SSF IOL managed aphakia and regular corneal astigmatism, achieving stable positioning, low misalignment, improved visual acuity, reduced refractive astigmatism, and preserved optical quality at 6 months.

Aphakia in the absence of capsular support remains a challenging surgical scenario, and several secondary intraocular lens (IOL) implantation strategies have been proposed, including iris-claw fixation, scleral-sutured IOLs, and sutureless flanged intrascleral fixation.^1–3^ Although each technique has specific advantages, they also present limitations related to surgical complexity, long-term fixation stability, and refractive predictability.

As refractive goals have increasingly become a central focus of modern cataract and anterior segment surgery, the management of preexisting corneal astigmatism has gained particular importance in aphakic patients. Although single-piece acrylic IOLs can be secured using transscleral fixation, long-term data show that a substantial proportion of these eyes experience clinically significant residual refractive astigmatism, which may compromise visual rehabilitation.^4–6^

The FIL–SSF IOL (Soleko SPA) is a single-piece, foldable hydrophilic acrylic IOL specifically designed for sutureless transscleral fixation through a pair of T-plug anchors.^7,8^ This system enables knot-free, stable fixation while avoiding complications associated with sutures, such as late degradation, erosion, and instability. Early clinical evidence has shown favorable safety and performance characteristics, including reliable self-centration, low tilt, and stable positioning.^9–11^ Furthermore, because the IOL is inserted through a small corneal incision, it minimizes surgically induced astigmatism when compared with traditional scleral-sutured approaches.

Given the high prevalence of corneal astigmatism in aphakic eyes without capsular support, a toric version of the FIL–SSF IOL (T FIL SSF) was developed to allow simultaneous correction of aphakia and astigmatism. However, clinical data on the refractive performance of this toric design remain limited.^12^

The purpose of this study was therefore to evaluate the visual, refractive, and aberrometric outcomes of T FIL–SSF IOL implantation in aphakic eyes with regular corneal astigmatism and no capsular support.

## METHODS

This multicenter, prospective observational case series included aphakic patients who underwent implantation of a T FIL–SSF IOL. The study was conducted at Ospedale Piero Palagi, Azienda USL Toscana Centro, and Azienda Ospedaliera Universitaria Careggi, Florence, Italy. Institutional review board approval was obtained, and all procedures adhered to the tenets of the Declaration of Helsinki. Written informed consent was obtained from all patients. Surgeries were performed between January 2024 and February 2025.

### Eligibility Criteria

Eligible eyes included aphakia secondary to crystalline lens subluxation or luxation, late in-the-bag IOL dislocation, or complicated cataract surgery. Eyes were required to have regular corneal astigmatism greater than 1 diopter (D), normal central corneal topography, and corneal higher-order aberrations less than 0.5 µm (4 mm pupil). Eyes with irregular corneal astigmatism—defined as principal meridians forming a skewed angle greater than 20 degrees—as well as those with corneal opacities, uncontrolled glaucoma, or retinal or optic nerve disease likely to limit visual potential were excluded.

### Preoperative Examination

All patients underwent a comprehensive ophthalmic evaluation including uncorrected distance visual acuity (UDVA), corrected distance visual acuity (CDVA) (Snellen), manifest refraction, and slitlamp biomicroscopy. Axial length (AL) was measured using optical biometry: AL-Scan (Nidek Co., Ltd.) at Ospedale Piero Palagi and IOLMaster 500 (Carl Zeiss Meditec AG) at Careggi Hospital. Corneal tomography and endothelial cell count were performed at both centers using the MS-39 anterior segment optical coherence tomography (AS-OCT) (Costruzione Strumenti Oftalmici) and the Perseus endothelial specular microscope (Costruzione Strumenti Oftalmici), respectively. Spherical and cylindrical IOL power calculations were provided by the manufacturer.

### Surgical Technique

In all patients, the 0- to 180-degree reference axis was marked at the slitlamp before surgery. A 25-gauge pars plana vitrectomy was performed using the Constellation Vision System (Alcon Laboratories, Inc.) under retrobulbar anesthesia.

In cases of crystalline lens subluxation or luxation, lens extraction was performed by phacoemulsification or pars plana lensectomy according to intraoperative findings. Similarly, in eyes with subluxated IOLs, the entire IOL–capsular bag complex was folded and explanted through a 2.4 mm corneal tunnel.

A limited conjunctival peritomy was followed by creation of 2 partial-thickness scleral grooves (3 mm) positioned 180 degrees apart and located 3 mm from the limbus. From each groove, a 1 mm scleral pocket was dissected toward the limbus to accommodate plug fixation. Two sclerotomies were created 2 mm posterior to the surgical limbus at the predetermined axis.

The T FIL–SSF IOL was inserted through the 2.4 mm corneal tunnel. The leading plug was grasped using 25-gauge crocodile-tip forceps passed through the sclerotomy and externalized beneath the scleral pocket. The trailing plug was externalized using the handshake technique, ensuring centration without further manipulation. The conjunctival wound was sealed with fibrin glue, whereas the corneal tunnel required no sutures. All surgeries were performed by 3 experienced surgeons (F.B., L.D., and F.G.). Video 1 summarizes the key surgical steps.

### Postoperative Examination

Patients were examined at postoperative days 1 and 7, and at 1 month, 3 months, and 6 months. At each visit, UDVA, CDVA, and manifest refraction were recorded, and postoperative complications were documented. At 6 months, vector astigmatism analysis, wavefront aberrometry, and IOL alignment were assessed.

### Astigmatism Vector Analysis

Double-angle plots of preoperative corneal astigmatism and postoperative refractive astigmatism were generated using the ASCRS Astigmatism Double-Angle Plot Tool (v. 1.3) downloaded from https://ascrs.org/tools/astigmatism-double-angle-plot-tool.^13^ Target-induced astigmatism (TIA) was defined as the vector difference between the intended postoperative refractive astigmatism and the preoperative keratometric astigmatism. Surgically induced astigmatism (SIA) represented the vector change between preoperative keratometric astigmatism and postoperative refractive astigmatism. The total prediction error, calculated as the vector difference between SIA and TIA, quantified the degree of overcorrection or undercorrection.

### Aberrometric Wavefront Analysis

Wavefront analysis was performed using the MS-39 AS-OCT and the Osiris aberrometer (Costruzione Strumenti Oftalmici) after pharmacologic dilation with 0.5% phenylephrine. Three consecutive measurements were acquired to ensure reproducibility. The MS-39 was used to quantify corneal aberrations, accounting for both anterior and posterior corneal surfaces. Total ocular aberrations were assessed with the Osiris aberrometer, and the corneal component derived from the MS-39 was subtracted from the total measurement to obtain the internal (lenticular) aberration profile.

Astigmatism (Z_2_^+2^ and Z_2_^−2^) and higher-order aberrations (HOAs) were quantified separately for the corneal, internal, and total optical components within a 4 mm pupil. An internal software application (Toric IOL Assistant, Phoenix Software v. 4.1.6.0) was then used to evaluate ocular toricity, including both corneal and internal components, and to calculate the degree of IOL misalignment, defined as the absolute angular difference between the internal astigmatism axis (IOL customized astigmatism axis) and the corneal astigmatism axis (Supplemental Material, available at http://links.lww.com/JRS/B614).

### Statistical Analysis

Descriptive statistics were reported as mean ± SD, with visual acuity converted to logMAR for analysis. The Spearman correlation coefficient was used to explore the pattern of correlation between variables. Preoperative and postoperative data were analyzed using generalized linear models with a robust (sandwich or Huber-White) variance estimator to account for correlated data within participant. A *P* value of less than 0.05 was considered statistically significant. All analyses were conducted using StataNow 19.5 (Statacorp LLC).

## RESULTS

### Population

Of the 17 eyes that underwent T FIL–SSF IOL implantation, 15 eyes from 12 patients (8 men and 4 women) completed the 6-month follow-up and were included in the analysis. One eye was excluded because of postoperative retinal detachment and 1 because of loss to follow-up.

The cohort included 7 eyes (46.7%) with ectopia lentis associated with cataract (5 eyes from 3 patients with Marfan syndrome and 2 eyes without underlying collagenopathy), 4 eyes (26.7%) with crystalline lens subluxation or luxation after blunt trauma, 2 eyes (13.3%) aphakic after complicated cataract surgery, and 2 eyes (13.3%) with late in-the-bag IOL dislocation.

Mean age was 48.1 ± 21.2 years. Mean axial length was 23.43 ± 1.65 mm. Mean keratometry values were 41.89 ± 2.07 D (Kflat) and 44.10 ± 2.02 D (Ksteep). The implanted IOLs had a mean spherical power of 22.71 ± 5.23 D and a mean cylindrical power of 2.23 ± 0.59 D (Table 1).

**Table 1.** Demographic data

Variable	Value
Eyes included (n)	15
Patients (n)	12
Sex (M/F)	8/4
Age (y), mean ± SD	48.1 ± 21.2
Ectopia lentis, n (%)	7 (46.7%)
Posttraumatic subluxation/luxation, n (%)	4 (26.7%)
Aphakia after complicated cataract surgery, n (%)	2 (13.3%)
Late in-the-bag IOL dislocation, n (%)	2 (13.3%)
Axial length (mm), mean ± SD	23.43 ± 1.65
Kflat (D), mean ± SD	41.89 ± 2.07
Ksteep (D), mean ± SD	44.10 ± 2.02
Spherical IOL power (D), mean ± SD	22.71 ± 5.23
Toric IOL cylinder power (D), mean ± SD	2.23 ± 0.59

### Surgical and Refractive Outcomes

Table 2 summarizes surgical and refractive outcomes.

**Table 2.** Surgical and refractive outcomes

Variable	Preoperative Mean ± SD	Postoperative Mean ± SD	*P* value
UDVA (logMAR)	0.96 ± 0.25	0.19 ± 0.10	<.01
CDVA (logMAR)	0.41 ± 0.32	0.04 ± 0.05	<.01
SE (D)	3.44 ± 8.16	−0.23 ± 0.63	<.01
Refractive astigmatism (D)	2.57 ± 0.93	0.70 ± 0.51	<.01
ECC (cells/mm^2^)	2701.5 ± 636.3	2442.5 ± 672.6	<.01

ECC = endothelial cell count; SE = spherical equivalent

**Figure 1.**
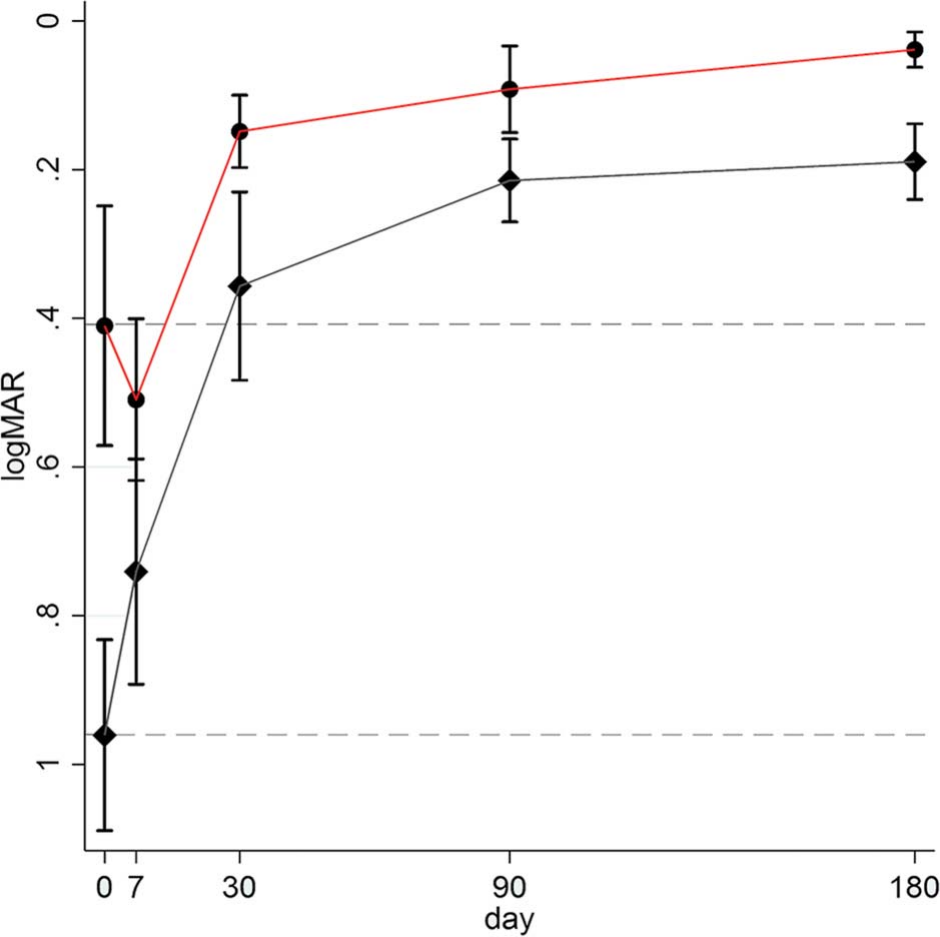
Mean CDVA (circles) and UDVA (diamonds) expressed as logMAR.

Mean UDVA improved significantly from 0.96 ± 0.25 to 0.19 ± 0.10 logMAR at 6 months (*P* < .01). Mean CDVA improved from 0.41 ± 0.32 to 0.04 ± 0.05 logMAR (*P* < .01) (Figure 1).

Endothelial cell count decreased from 2701.5 ± 636.3 to 2442.5 ± 672.6 cells/mm^2^ at the 6-month follow-up (*P* < .01).

The mean preoperative spherical equivalent (SE) was 3.44 ± 8.16 D, while the mean postoperative SE was −0.23 ± 0.63 D (*P* < .01).

Mean absolute refractive astigmatism significantly decreased from 2.57 ± 0.93 D preoperatively to 0.70 ± 0.51 D postoperatively (*P* < .01).

**Figure 2.**
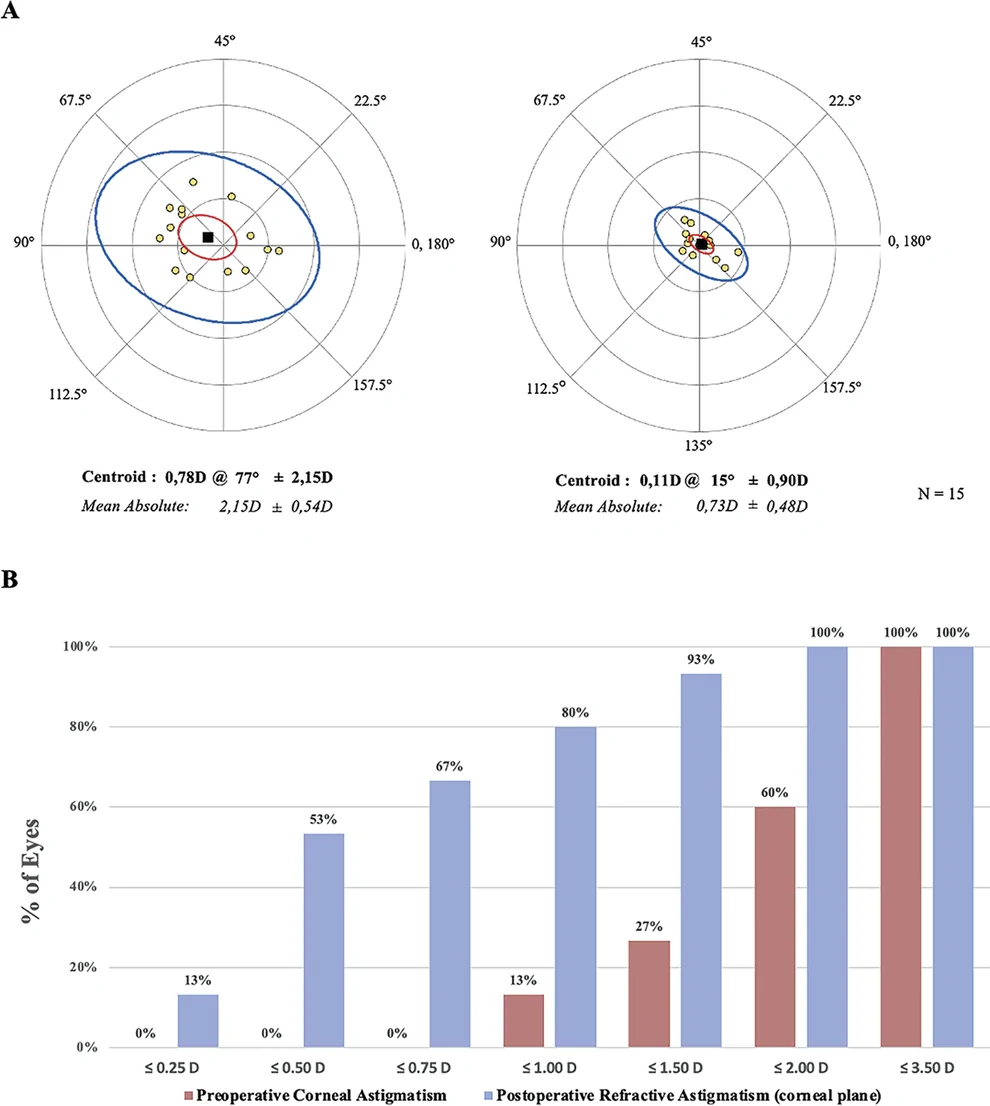
Vectoral analysis of astigmatic correction. *A*: Double-angle plots of the preoperative corneal and postoperative refractive astigmatism with centroids (N = 15). *B*: Cumulative histogram illustrating magnitude of preoperative corneal and postoperative refractive astigmatism, vertexed to the corneal plane (N = 15).

Mean absolute preoperative corneal astigmatism was 2.15 ± 0.54 D (centroid: 0.78 D @ 77 degrees ± 2.15 D). At 6 months, mean postoperative refractive astigmatism decreased to 0.73 ± 0.48 D (centroid: 0.11 D @ 15 degrees ± 0.9 D), with 12 of 15 eyes (80%) achieving ≤1.0 D of residual refractive astigmatism (Figure 2A and B).

The mean SE prediction error was −0.12 ± 0.54 D. Eleven of 15 eyes (73.3%) were within ±0.50 D, 13 of 15 eyes (86.7%) within ±1.00 D, and all eyes (100%) within ±1.25 D of the intended refractive target (Table 3).

**Table 3.** Distribution of eyes within target refraction

Parameter	Value
Eyes within ±0.50 D	11/15 (73.3%)
Eyes within ±1.00 D	13/15 (86.7%)
Eyes within ±1.25 D	15/15 (100%)
Mean SE prediction error (D)	−0.12 ± 0.54

SE = spherical equivalent

Postoperative complications included 1 case of transient vitreous hemorrhage and 1 case of macular edema at 3 months; both resolved with conservative management.

### Wavefront Aberrometry Analysis

Mean corneal astigmatism was 0.79 ± 0.31 µm, mean internal astigmatism was 0.78 ± 0.28 µm, and total astigmatism was 0.36 ± 0.28 µm (*P* < .05).

**Figure 3.**
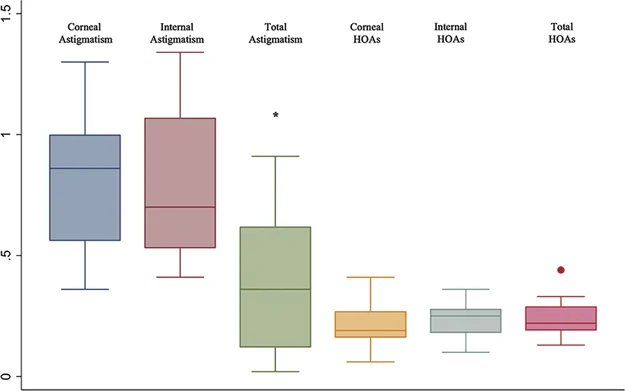
Boxplot comparison of corneal, internal, and total astigmatism and HOAs at the 6-month follow-up. *P < .05 comparison between corneal and total astigmatism.

Corneal HOAs measured 0.22 ± 0.10 µm; internal HOAs measured 0.23 ± 0.07 µm. Total HOAs were 0.24 ± 0.08 µm, with no significant difference from corneal HOAs (*P* > .05) (Figure 3).

Mean IOL misalignment was 3.33 ± 3.42 degrees; a significant positive correlation was found between misalignment and residual refractive astigmatism (ρ = 0.71, *P* < .01).

## DISCUSSION

This study presents the first clinical and refractive outcomes of the T FIL SSF IOL, evaluated in a cohort of eyes requiring secondary implantation due to inadequate capsular support and the presence of significant corneal astigmatism.

The implantation of T FIL SSF IOL resulted in significant improvements in both UDVA and CDVA at 6 months; these outcomes should be interpreted in light of the study population, which included eyes with aphakia or lens/IOL dislocation and subluxated crystalline lenses associated with cataract, without underlying ocular comorbidities limiting visual potential.

The mean absolute postoperative prediction error SE observed in our cohort was comparable with values previously reported for the nontoric (monofocal) FIL SSF IOL, which ranged from 0.24 ± 0.81 to 0.70 ± 2.00 D.^9,11,14–16^ Reported refractive accuracy with this nontoric lens design, using IOL power calculation formulas that do not rely on anterior chamber depth measurements, indicates that 56% to 64% of eyes achieve prediction errors within ±0.50 D and 69% to 72% within ±1.00 D, with long-term series reporting up to 77% of eyes within ±1.00 D.^11,17^ In our cohort, the slightly lower absolute mean prediction error, together with the higher proportion of eyes within ±0.50 D of the intended refractive target, likely reflects the refractive advantage of astigmatic correction provided by the T FIL SSF design.

Vector analysis demonstrated a consistent reduction in astigmatism after surgery with a mean absolute refractive astigmatism of 0.73 ± 0.48 D, with a centroid of 0.11 D @ 61 degrees ± 0.9 D, indicating a marked reduction in both magnitude and directional dispersion. These astigmatic outcomes are consistent with those reported for other approaches that adapt in-the-bag toric C-loop IOLs for scleral fixation. Pan et al. reported a residual cylinder of 0.77 ± 0.54 D using the SN6AT toric IOL with sutured fixation, while Ward et al. and Swaminathan et al. found similar values using the MX60T IOL secured with Gore-Tex fixation.^18–20^ More recently, Zhang et al. described favorable results with a flanged scleral fixation of the Tecnis Toric II IOL, although their lower baseline astigmatism may partly explain the smaller postoperative cylinder.^21^

Although effective, many of these techniques require structural modification of the lens—such as haptic trimming—or the creation of a suture knot around the haptic, both of which have been associated with complications including iris chafing, persistent inflammation, lens tilt, and late mechanical instability. Gore-Tex–based fixation improves long-term stability, while flanged fixation eliminates suture-related issues; however, both techniques still require intraoperative rotational alignment to match the corneal astigmatic axis, increasing surgical complexity and variability. Furthermore, concerns persist regarding long-term fixation stability which are particularly relevant in toric IOLs.^22^

By contrast, the T FIL–SSF IOL incorporates the astigmatic axis directly within the optic and relies on 2 plug-based scleral fixation points, allowing a standardized implantation without intraoperative rotation. Previous studies of the monofocal FIL–SSF have reported stable centration and absence of late dislocation, suggesting that this fixation method inherently resists torsional displacement.^11^

In this study, misalignment was objectively assessed using pyramidal wavefront aberrometry, which allows direct evaluation of toric IOL orientation by differentiating corneal from internal optical components.^23,24^ The mean misalignment of 3.33 degrees aligns with the pooled mean absolute rotation of 2.36 degrees reported in a recent systematic review and meta-analysis.^25^ Importantly, unlike in-the-bag toric IOLs—where postoperative rotation is the primary contributor to misalignment—the plug-based design of the FIL–SSF likely limits true rotation. Misalignment in this context, therefore, reflects small deviations in the placement and symmetry of the scleral tunnels rather than postoperative rotational drift. As expected, a correlation was observed between misalignment and residual refractive astigmatism, consistent with previous evidence indicating that the refractive performance of toric IOLs is highly sensitive to rotational deviations.^26^

Aberrometric evaluation demonstrated a significant reduction in total astigmatism compared with corneal astigmatism, suggesting the contribution of the internal toric correction provided by the IOL. Conversely, total HOAs remained comparable with corneal HOAs, suggesting that internal optical aberrations introduced by the IOL were minimal. Notably, the magnitude of internal HOAs was comparable with values previously reported for the nontoric FIL SSF IOL at the same pupil diameter, suggesting that although tilt and decentration were not directly measured in this study, their impact is likely negligible.^16^

The study has limitations. The sample size was limited, reducing statistical power and limiting generalizability. The lack of a control group precluded direct comparison with alternative fixation techniques. Although the 6-month data were encouraging, a longer follow-up is needed to further characterize refractive stability over time.

Despite these limitations, the present findings indicate that the T FIL–SSF IOL provides effective correction of aphakia and regular corneal astigmatism with stable sutureless fixation and preserved optical quality. Future controlled studies with larger samples and longer follow-up will help define the comparative role of this technique among existing options for secondary IOL implantation in eyes lacking capsular support with significant corneal astigmatism.

**WHAT WAS KNOWN**

- Aphakia without capsular support presents substantial surgical and refractive challenges, especially in eyes with significant corneal astigmatism.
- The FIL–SSF IOL platform has demonstrated stable sutureless transscleral fixation, with favorable centration and low tilt in its monofocal version.
- Achieving reliable toric correction in secondary IOL fixation is difficult because of risks of rotation, haptic modification, or suture-related instability.

**WHAT THIS PAPER ADDS**

- This study provides the first clinical data on the toric FIL–SSF IOL in eyes with inadequate capsular support and significant corneal astigmatism.
- Toric FIL–SSF IOL implantation was associated with improved visual acuity and reduced refractive astigmatism, with low higher-order aberrations at 6 months.
- Low misalignment values suggest that this platform may mitigate rotational instability, a major limitation of other toric secondary IOL techniques.
